# Usefulness of lung ultrasound for early detection of hospital-acquired pneumonia in cardiac critically ill patients on venoarterial extracorporeal membrane oxygenation

**DOI:** 10.1186/s13613-022-01013-9

**Published:** 2022-05-21

**Authors:** Jean Pasqueron, Pauline Dureau, Gauthier Arcile, Baptiste Duceau, Geoffroy Hariri, Victoria Lepère, Guillaume Lebreton, Jean-Jacques Rouby, Adrien Bouglé

**Affiliations:** 1 Sorbonne Université, GRC 29, AP-HP, DMU DREAM, Department of Anesthesiology and Critical Care, Institute of Cardiology, Pitié-Salpêtrière Hospital, 47-83 boulevard de l’Hôpital, 75651 Paris Cedex 13, France; 2 Sorbonne Université, Department of Cardiac Surgery, Institute of Cardiology, Pitié-Salpêtrière Hospital, Paris, France; 3 Sorbonne Université, GRC 29, AP-HP, DMU DREAM, Department of Anesthesiology and Critical Care, Multidisciplinary Intensive Care Unit, Pitié-Salpêtrière Hospital, Paris, France

**Keywords:** Venoarterial extracorporeal membrane oxygenation, Hospital-acquired pneumonia, Lung ultrasound, Doppler color lung ultrasound, Dynamic air bronchogram, Color Doppler intrapulmonary flow, Intensive care unit

## Abstract

**Background:**

Hospital-acquired pneumonia (HAP) is the most common and severe complication in patients treated with venoarterial extracorporeal membrane oxygenation (VA ECMO) and its diagnosis remains challenging. Nothing is known about the usefulness of lung ultrasound (LUS) in early detection of HAP in patients treated with VA ECMO. Also, LUS and chest radiography were performed when HAP was suspected in cardiac critically ill adult VA ECMO presenting with acute respiratory failure. The sonographic features of HAP in VA ECMO patients were determined and we assessed the performance of the lung ultrasound simplified clinical pulmonary score (LUS-sCPIS), the sCPIS and bioclinical parameters or chest radiography alone for early diagnosis of HAP.

**Results:**

We included 70 patients, of which 44 (63%) were independently diagnosed with HAP. LUS examination revealed that color Doppler intrapulmonary flow (*P* = *0.0000043*) and dynamic air bronchogram (*P* = *0.00024*) were the most frequent HAP-related signs. The LUS-sCPIS (area under the curve = 0.77) yielded significantly better results than the sCPIS (area under the curve = 0.65; *P* = *0.004*), while leukocyte count, temperature and chest radiography were not discriminating for HAP diagnosis.

**Discussion:**

Diagnosis of HAP is a daily challenge for the clinician managing patients on venoarterial ECMO. Lung ultrasound can be a valuable tool as the initial imaging modality for the diagnosis of pneumonia. Color Doppler intrapulmonary flow and dynamic air bronchogram appear to be particularly insightful for the diagnosis of HAP.

**Supplementary Information:**

The online version contains supplementary material available at 10.1186/s13613-022-01013-9.

## Background

Venoarterial extracorporeal membrane oxygenation (VA ECMO) is an effective rescue therapy providing temporary cardiac and respiratory support for patients with refractory cardiogenic shock. VA ECMO allows organ perfusion and oxygenation while awaiting myocardial recovery, cardiac transplantation or long-term mechanical circulatory support. Significant progress in device technology and in the management of critically ill patients have allowed an international expansion of the use of VA ECMO [1]. However, short-term mortality remains high, with an overall survival rate of 40% [2], mainly due to the very high incidence of complications, among which infections are predominant. According to recent studies, hospital-associated pneumonia (HAP) is the most frequent nosocomial infection among VA ECMO patients [3, 4]. Its occurrence has been consistently associated with a longer duration of ECMO support and mechanical ventilation, and an increased mortality [5]. However, early diagnosis of HAP in VA ECMO patients remains a daily challenge.

Point-of-care lung ultrasound (LUS) was demonstrated to be an effective tool in case of acute respiratory failure for ICU patients [6, 7], community-acquired pneumonia [8, 9], and ventilator-associated pneumonia [10, 11]. The LUS diagnosis of ventilator-associated pneumonia in intensive care units is more challenging in comparison with the diagnosis of community-acquired pneumonia in emergency departments due to the limited access to the mechanically ventilated patients and the high prevalence of atelectasis. However, several studies have demonstrated that the combination of LUS findings with other clinical markers could improve the diagnostic accuracy. This bioclinical and lung ultrasound approach has been successfully applied to diagnose postoperative pneumonia after cardiac surgery [12]. The substitution of chest radiography by color Doppler LUS (LUS-sCPIS) in the simplified Clinical Pulmonary Infection Score (sCPIS) improved the diagnostic accuracy for postoperative pneumonia in patients who underwent cardiac surgery with cardiopulmonary bypass. However, nothing has been published on the feasibility and the place of lung ultrasound in the array of tools to diagnose pneumonia in VA ECMO patients. When LUS is used in this population, sonographic features of patients without ECMO are sought.

The aim of this study was to determine the feasibility and usefulness of LUS in the early detection of HAP in cardiac critically ill patients under VA ECMO as well as assess its sonographic features.

## Methods

### Patient selection and study design

Patients assisted with VA ECMO, and who presented with acute respiratory failure during ECMO support between May 2018 and May 2020 were included. Acute respiratory failure was defined by at least one of the following criteria: the necessity to increase the fraction of delivered oxygen in the sweep gas by 20% for more than 6 h; the need to increase the inspired fraction of oxygen (FiO_2_) by 20% for more than 6 h in patients under mechanical ventilation; clinical signs of acute respiratory distress (cyanosis, pulse oximeter oxygen saturation (SpO_2_) less than 90%, tachypnea > 25 breaths/min, upper thoracic or intercostal in drawing), de novo dependence on high-flow nasal oxygen therapy or non-invasive ventilation and/or the need for orotracheal intubation. The study protocol complied with the Declaration of Helsinki and was approved by the CERAR (Ethics Committee of the Société Française d’Anesthésie et de Réanimation, IRB 00,010,254-2019-174).

### Pneumonia diagnosis

All patients admitted to our center receive a bundle of interventions to reduce the risk of ventilator-associated pneumonia (Additional file 4: Table S1). The criteria for the diagnosis of pneumonia were based on the guidelines for the management of adults with HAP [13, 14]. Clinical and radiological criteria were used to identify suspected pneumonia (≥ two criteria among fever > 38.5 °C, leukocytosis > 11.10^9^/L or leukopenia < 4.10^9^ /L, purulent tracheobronchial secretions and a new or persistent pulmonary infiltrate on bedside chest radiography). A positive quantitative culture of a lower respiratory tract sample was a main criterion to establish the diagnosis: either a positive bronchoalveolar lavage fluid (significant threshold ≥ 10^4^ colony-forming units (CFU) /ml) or a positive plugged telescopic catheter (significant threshold ≥ 10^3^ CFU/ml). The final diagnosis of pneumonia was retained, after microbiological results, when it was made by at least two of three independent physicians and it was rejected when ruled out by at least two of the three physicians, who were all blinded to LUS examinations.

### Measurement of intrapulmonary flow using Doppler with color-flow mapping

**Fig. 1:**
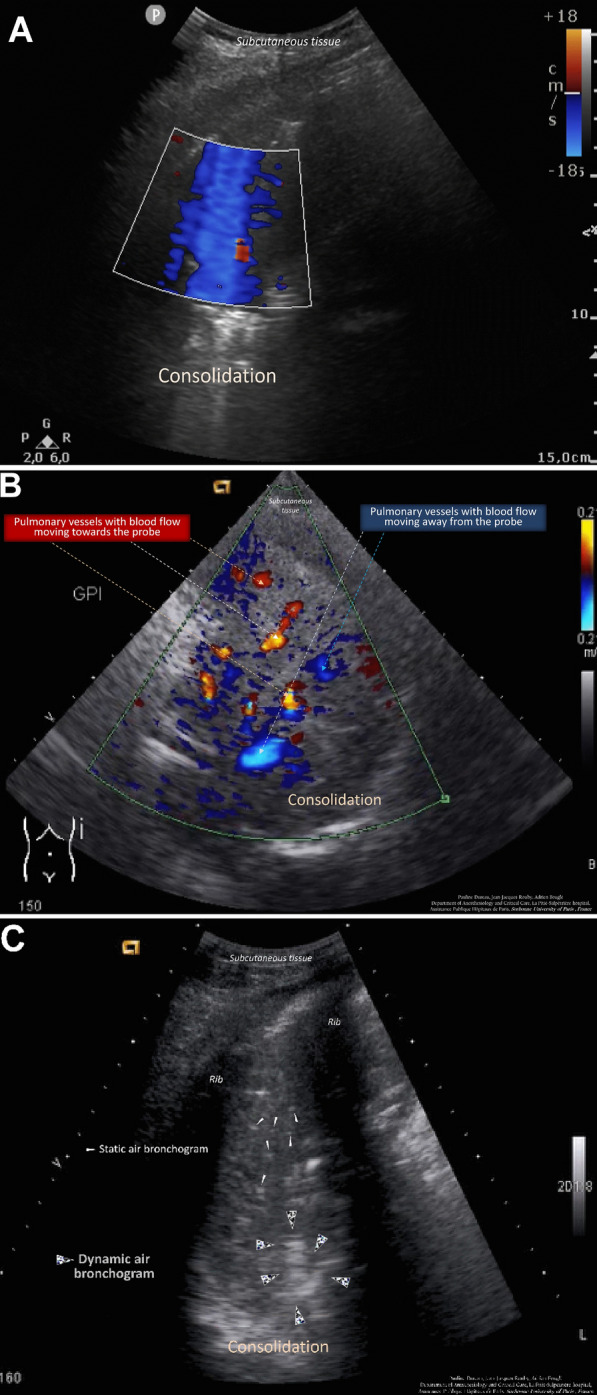
**A** Color Doppler lung ultrasound in the consolidated left lower lobe in a 54-year-old patient without pneumonia on venoarterial extracorporeal membrane oxygenation. The extracorporeal membrane oxygenation was initiated 2 days before and pneumonia was ruled out by a bronchoalveolar lavage retrieving 2.10^2^ orophayngeal flora. Color signals are diffuse and changing resulting from interferences caused by respiratory movements and heart beats. Corresponding to Video S1A (Additional File 1). **B** Color Doppler intrapulmonary flow detected in the consolidated right lower lobe of a 43-year-old patient with pneumonia on venoarterial extracorporeal membrane oxygenation. The extracorporeal membrane oxygenation was initiated 12 days before for a cardiogenic shock and pneumonia was confirmed by a bronchoalveolar lavage retrieving 10^4^ *Escherichia coli*. The blood flow in a vessel is seen as a color signal persisting in the same location during the respiratory cycle with a tubular, curvilinear, or branching distribution on real-time images. When blood flow signals are detected, pulse-wave Doppler can identify their spectral waveform.^19^ Corresponding to Video S1B (Additional File 2). **C** Dynamic air bronchogram in a 79-year-old patient with pneumonia on venoarterial extracorporeal membrane oxygenation. The extracorporeal membrane oxygenation was initiated 5 days before for a cardiogenic shock and pneumonia was confirmed by a bronchoalveolar lavage retrieving 6.10^6^ *Hafnia alvei*. Corresponding to Video S1C (Additional File 3)

Within consolidations, blood flow signals were detected using Doppler with color-flow mapping as previously described [15, 16]. To detect low velocity flow and avoid interference from respiratory movement and heart beats, the following parameters were selected: velocity of 0.21 m/s, Doppler angle of 0°–180°, and wall filter range 70–120 kHz. The color Doppler gain was increased until the background noise appeared as a colored “snowstorm” across the image, and was then backed off until only a few random specks remained visible (Fig. 1A and B). Additional movie files show in more details the use of color Doppler ultrasound without intrapulmonary flow (Additional File 1) and with intrapulmonary flow (Additional File 2), corresponding, respectively, to Fig. 1A and B. A color Doppler intrapulmonary flow was defined as a persistent area of color signal with a tubular, curvilinear, or branching distribution persisting during the respiratory cycle.

### Lung ultrasound, sCPIS, and LUS-sCPIS

Bedside lung ultrasound was performed on the day of inclusion by an experienced physician for pulmonary ultrasound (performance of more than 100 pulmonary ultrasound examinations). The images were recorded and then retrospectively analyzed in a blinded fashion. Lung ultrasound examination and the image analysis were standardized. The areas to be examined were divided into 12 regions, i.e., 6 regions per hemithorax as previously described [17]. Ultrasound signs suggestive of pneumonia were the presence of juxta pleural consolidations associated or not with B lines [11, 18], one or more pulmonary consolidations, the presence of a color Doppler intrapulmonary flow [8], and/or the presence of static or dynamic air bronchogram within these consolidations (Fig. 1C and Additional File 3) [18]. To assess the loss of aeration in each lung zone, a numeric value was assigned according to the most severe lung ultrasound finding detected in the corresponding examined region. The lung ultrasound aeration score was calculated as the sum of the numeric values assigned to each examined region, ranging from 0 to 36. The standardized LUS protocol used in this study is detailed in supplemental data (Additional file 4: Table S2).

To assess to what extent LUS is as sensitive as chest radiography to screen VA ECMO patients for HAP, we used the simplified Clinical Pulmonary Infection Score (sCPIS) (Additional file 4: Table S3) described by Luna et al. [19] and the Lung ultrasound-simplified Clinical Pulmonary Infection Score (LUS-sCPIS) described previously in cardiac surgery patients (Additional file 4: Table S4). In this score, the "chest radiography" criterion is replaced by an ultrasound criterion, the presence or not of a color Doppler intrapulmonary flow within a consolidation. The presence of a color Doppler intrapulmonary flow counts for two points [12].

### Statistical analysis

By estimating the prevalence of pneumonia at 50% in patients on VA ECMO [3, 4], a sample size of 70 patients was required for detecting a change in the percentage value of sensitivity of LUS-sCPIS from 50 to 80%, with an 80% power at 0.05 alpha risk. Quantitative variables are presented by their median and quartiles (25th and 75th percentile). Qualitative variables are represented by their numbers and percentages. A univariate analysis was performed to compare the two groups, using the Mann–Whitney Wilcoxon test for quantitative variables and the Chi^2^ or Fisher test for qualitative variables. Sensitivity, specificity, positive predictive value, negative predictive value and area under the receiver operating characteristic (ROC) curves were calculated to determine the diagnostic performance of sCPIS and LUS-sCPIS. The pulmonary ultrasound parameters were studied independently: dynamic air bronchogram, color Doppler intrapulmonary flow or a combination of these two parameters.

The diagnostic performances of the sCPIS and LUS-sCPIS were described using ROC curves. The areas under the curves (AUCs) of the ROC curves were compared using a non-parametric Delong test. Due to the small number of missing data, no imputation was performed. The significance threshold was chosen at 5%. The analyses were performed using R statistical software (version 3.6.1; R Foundation for Statistical Computing, Vienna, Austria).

## Results

Between May 2018 and May 2020, 70 cardiac critically ill patients assisted with VA ECMO hospitalized in the cardiac ICU were included and evaluated. Diagnosis of HAP was established in 44 patients (63%), all of whom had a positive microbiological sample. The study population consisted of 70% men and 30% women. The median age was 63.1 years [56.0–68.1]. Fifty patients had cardiogenic shock after cardiac surgery (71.4%). Of these patients, 18 had undergone heart transplantation, 15 valve surgery, 5 coronary bypass surgery, and 6 combined surgery. The median CBP duration was 138 min and the median preoperative Euroscore 2 was 20.5 [3.66–32.0] [20]. The 20 patients assisted for medical reasons had refractory cardiogenic shock related to ischemic heart disease (*N* = 17) or myocardial rejection after heart transplantation (*N* = 3). Fifty-nine patients were on mechanical ventilation on the day of inclusion (84%). Five patients received de novo treatment with non-invasive ventilation or high-flow nasal oxygen therapy without the need for intubation afterward. The median time from ECMO initiation to inclusion was 5 days [2.25–7.00]. The rate of patients on antibiotic therapy at inclusion was higher in the pneumonia group (75% vs. 46%, *P* = *0.015*), as was the median total duration of mechanical ventilation (16.0 days [9.00–30.5] vs. 7.5 days [5.00–16.2], *P* = *0.002*). Other descriptive data are presented in Table 1.

**Table 1:** Patient characteristics grouped by the presence or absence of pneumonia

Patient characteristics	Pneumonia		*P*
	No (*n* = 26)	Yes (*n* = 44)	
Age (y)	63.5 [57.5 – 67.3]	62.6 [53.8 – 68.5]	0.45
Male (%)	18 (69)	31 (70)	0.91
Comorbidities			
BMI	27.5 [25.0 – 30.8]	26.0 [23.8 – 29.5]	0.45
Euroscore 2	15.5 [5.99 – 25.0]	21.1 [2.56 – 32.5]	0.86
SOFA	12.0 [11.0 – 16.0]	11.5 [10.0 – 14.0]	0.2
Diabetes mellitus (%)	9 (35)	9 (20)	0.19
COPD (%)	4 (15)	4 (9)	0.46
Reason for admission			
Heart surgery	17 (65)	33 (75)	0.46
Heart transplantation	7 (27)	11 (25)	0.86
CBP length (min)	120 [99 – 142]	152 [100 – 207]	0.099
ICU stay before inclusion (d)	3.50 [2.00 – 5.75]	5.00 [3.00 – 7.25]	0.073
Days of ECMO before inclusion	3.50 [2.00 – 6.00]	5.00 [3.00 – 7.25]	0.073
ECMO output (L/min)	4.0 [3.52 – 4.52]	4.0 [3.0 – 4.5]	0.4
FmO_2_	0.7 [0.6 – 0.8]	0.7 [0.5 – 0.8]	0.45
Mechanical ventilation (%)	22 (85)	37 (84)	1
FiO_2_	0.6 [0.4 – 0.9]	0.6 [0.5 – 0.7]	0.69
Vasopressor support (%)	22 (85)	28 (64)	0.06
Inotropic support (%)	15 (58)	24 (55)	0.8
Dobutamine (µg/kg/min)	3.85 [0 – 5.0]	2.75 [0 – 5.0]	0.56
Antibiotics at inclusion (%)	12 (46)	33 (75)	0.015
Leucocyte count (10^9^/l)	13.3 [8.84 – 17.6]	12.2 [9.36 – 17.0]	0.55
Duration of mechanical ventilation (d)	7.50 [5.0 – 16.2]	16.0 [9.00 – 30.5]	0.02
Days of ECMO (d)	13.0 [7.75—20.2]	15.0 [10.0—26.8]	0.19
ICU mortality (%)	14 (58)	19 (43)	0.23

Values are given as the median [IQR] or number (%)

*BMI* body mass index, *COPD* chronic obstructive pulmonary disease, *CPB* cardiopulmonary bypass, *ICU* intensive care unit, *ECMO* extracorporeal membrane oxygenation, *FiO*_*2*_ fraction of inspired oxygen, *FmO*_*2*_ fraction of delivered oxygen in the sweep gas on VA ECMO, *SOFA* Sequential Organ Failure Assessment

The only discriminating clinical criteria for the diagnosis of pneumonia were the abundance of tracheal secretions and their purulent aspect. Tracheal secretions were described as abundant in 52% of the patients with pneumonia (*N* = 23) versus 19% (*N* = 5) in the group without pneumonia (*P* = *0.0064*). Tracheal secretions were scanty in 23% (*N* = 10) of patients with pneumonia versus 58% (*N* = 15) of patients without pneumonia (*P* = *0.00016*). Secretions were purulent in 43% (*N* = 19) of patients with pneumonia versus 19% (*N* = 5) of patients without pneumonia (*P* = *0.041*). Temperature, leukocyte count and chest radiography findings were not significantly different between the two groups (Additional file 4: Table S5).

**Fig. 2:**
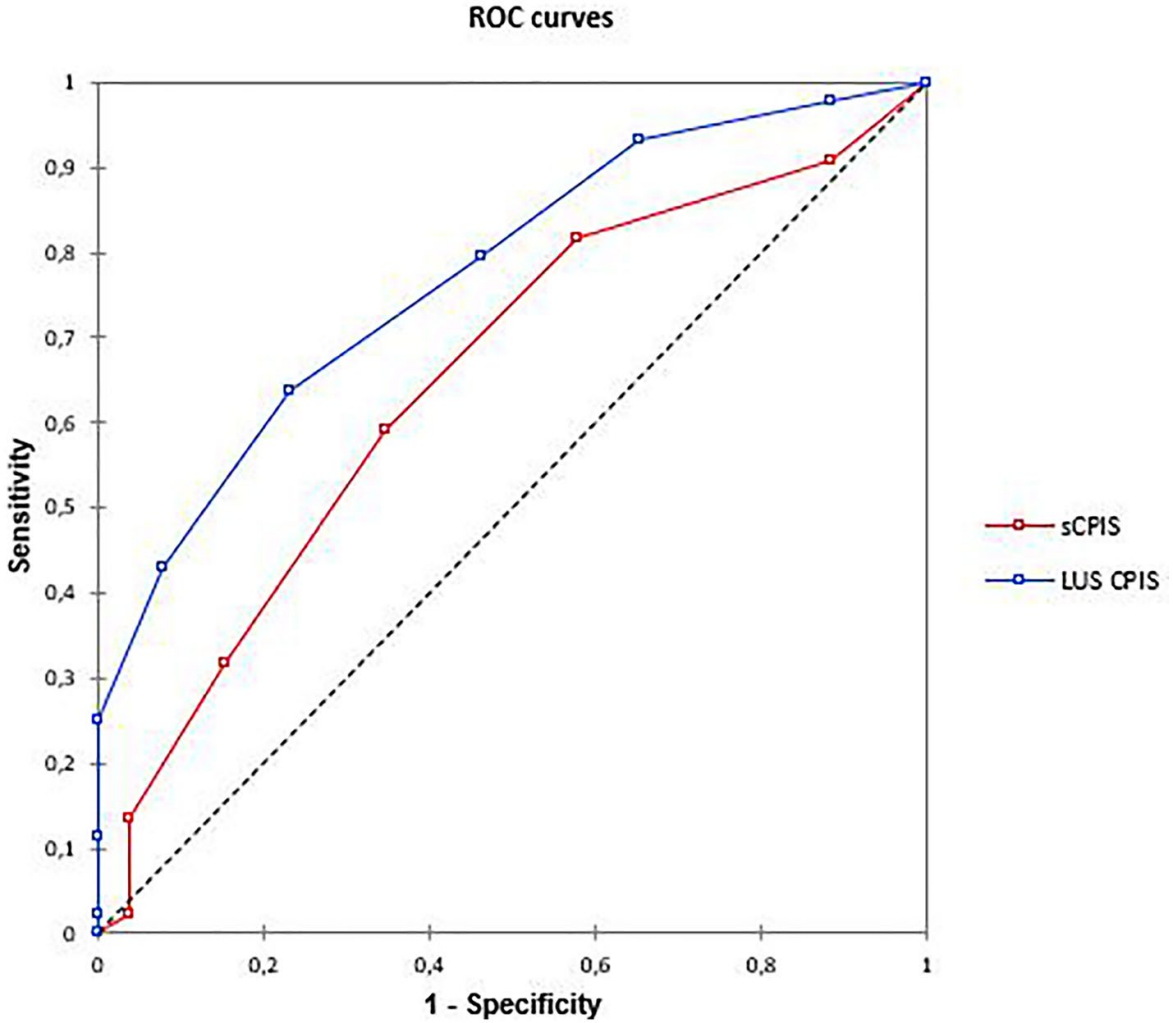
Receiver operating characteristic (ROC) curves of simplified Clinical Pulmonary Infection Score and Lung ultrasound-simplified Clinical Pulmonary Infection Score. LUS = lung ultrasound; sCPIS = simplified Clinical Pulmonary Infection Score

Lung consolidations were found in almost all patients (94%). In 25% of patients, pulmonary edema, defined as more than two examined regions characterized by multiple coalescent vertical B lines, was observed on top of consolidation. Pulmonary edema was more frequent in patients with pneumonia (30%) than in patients without pneumonia (20%), but the difference did not reach statistical significance (*P* = *0.18*). The extension of consolidation was a discriminating criterion: 43% of patients with pneumonia had more than three examined regions characterized by 3 versus 12% of patients without pneumonia (*P* = *0.006*). A color Doppler intrapulmonary flow present within a consolidation (Fig. 1B) was found in 47% of the patients and was significantly associated with the diagnosis of pneumonia (66% in patients with pneumonia versus 15% in patients without pneumonia, *P* = 0.0000043). An air bronchogram was visualized in 47 patients (67%). The dynamic nature of the air bronchogram was found in 50% of the patients with pneumonia (*N* = 20) and only in a single patient in the group without pneumonia (*P* = *0.00024*). The median lung ultrasound aeration score was 15 [11–20] with significantly more aeration loss in the pneumonia group (LUS = 17 [12–20] versus 12 [10–14], *P* = *0.007*) (Table 2). In the group of patients with pneumonia, 64% had a combination of color Doppler intrapulmonary flow and dynamic air bronchogram, compared to 15% of patients in the group without pneumonia (*P* = 0.000009). The combination of a color Doppler intrapulmonary flow and a dynamic air bronchogram yielded an AUC of 0.76 for the diagnosis of pneumonia (Table 3). As shown in Fig. 2, the diagnostic performance of LUS-sCPIS, was significantly better than that of sCPIS: AUC 0.77 [0.67 to 0.88] and AUC 0.65 [0.52 to 0.78], respectively (*P* = *0.004*).

**Table 2:** Comparison of lung ultrasound patterns between groups with and without pneumonia

Lung ultrasound patterns	Total (*n* = 70)	Pneumonia		*P*
		No (*n* = 26)	Yes (*n* = 44)	
Consolidation (%)	66 (94)	25 (96)	42 (95)	1
Unilateral	16 (23)	10 (38)	7 (16)	0.072
Bilateral	50 (61)	15 (58)	35 (80)	0.051
Color Doppler intrapulmonary flow (%)	33 (47)	4 (15)	29 (66)	0.0000043
Air bronchogram (%)				
Static	26 (37)	10 (38)	16(36)	0.7
Dynamic	21 (30)	1 (4)	20(45)	0.00024
Color Doppler intrapulmonary flow + dynamic air bronchogram (%)	32 (46)	4 (15)	28 (64)	0.000009
Juxta pleural consolidation (%)	41 (59)	12 (29)	29 (71)	0.1
Lung ultrasound aeration score	15 [11 – 20]	12 [10 – 14]	17 [12 – 20]	0.007

Quantitative variables are expressed as median [interquartile range] and qualitative variables as number and percentage (%)

**Table 3:** Diagnostic accuracy of lung ultrasound signs

	Se	Sp	PPV	NPV	AUC
Color Doppler intrapulmonary flow	0.66 [0.51 – 0.78]	0.85 [0.66 – 0.94]	0.88 [0.77 – 0.99]	0.60 [0.44 – 0.75]	0.75 [0.65 – 0.85]
Dynamic air bronchogram	0.46 [0.32 – 0.60]	0.96 [0.79 – 1.00]	0.95 [0.86 – 1.00]	0.51 [0.37 – 0.65]	0.71 [0.66 – 0.79]
LUS-sCPIS	0.64 [0.49 – 0.76]	0.77 [0.56 – 0.89]	0.82 [0.61 – 0.90]	0.56 [0.39 – 0.72]	0.77 [0.67 – 0.88]
sCPIS	0.59 [0.44 – 0.72]	0.65 [0.46 – 0.80]	0.74 [0.60 – 0.88]	0.49 [0.33 – 0.64]	0.65 [0.52 – 0.78]

LUS-sCPIS and sCPIS in the detection of VAP

*Se* sensivity, *Sp* specificity, *PPV*  positive predictive value, *NPV*  negative predictive value, *AUC*  area under the curve, *sCPIS* simplified Clinical Pulmonary Infection Score, *LUS-sCPIS* Lung ultrasound-simplified Clinical Pulmonary Infection Score, where the "chest radiography" criterion is replaced by the presence or not of a color Doppler intrapulmonary flow within a consolidation, visualized using color Doppler

## Discussion

This study, performed in cardiac critically ill patients on VA ECMO, yielded several findings. Regarding the sonographic features of HAP in this population, we found that: (1) consolidation of lower lobes was present in 94% of patients with acute respiratory failure, (2) diffuse pulmonary edema was present on top of consolidation in 30% of patients with pneumonia and in 15% of patients without pneumonia, (3) the presence of a color Doppler intrapulmonary flow or dynamic air bronchogram within consolidations was significantly associated with the diagnosis of pneumonia. Regarding the usefulness of LUS in the early detection of HAP, we highlighted that: (1) bedside chest radiography had poor performance to assess for the presence of pneumonia in patient with VA ECMO, (2) the presence of a color Doppler intrapulmonary flow or dynamic air bronchogram within consolidations was significantly associated with the presence of pneumonia, and (3) the diagnostic performance of the Lung Ultrasound Score sCPIS, in which color Doppler replaces bedside chest radiography, was higher than the conventional sCPIS. This last point suggests that LUS could outperformed chest radiography. Our results confirm the usefulness of LUS examination and LUS-SCPIS for accurate detection of pneumonia in VA ECMO patients and enrich previous literature about diagnostic accuracy of LUS-sCPIS [12, 21].

### Components of sCPIS and LUS-sCPIS: diagnostic value in patients on VA ECMO

HAP diagnosis is known to be difficult in VA ECMO patients [22]. We hypothesized that the criteria usually used to diagnose pneumonia may not be discriminating in VA ECMO patients. Indeed, the ECMO itself, or the circumstances in which it was initiated (post cardiotomy, cardiogenic shock, cardiac arrest) may be associated with a systemic inflammatory response, and the interpretation of chest radiography is complicated by the frequent presence of cardiogenic pulmonary edema. As expected, criteria such as body temperature and leucocyte count were comparable in both groups. The level of vasopressor support was not discriminating. In clinical practice we did not use any inflammatory marker such as procalcitonin for several reasons. Firstly, procalcitonin cannot reliably differentiate sepsis from other non-infectious causes of systemic inflammatory response syndrome in critically ill adult patients [23]. Secondly, the international guidelines for the management of sepsis recommends against the use of procalcitonin in sepsis/septic shock to initiate antibiotic therapy [24]. Thirdly, after heart transplantation, procalcitonin has shown to be increased in the early postoperative course, with a peak on the 3rd day, for all patients with or without infectious complications [25]. For these reasons, we do not routinely use procalcitonin to establish the diagnosis of infection. In any event, the difficulty of establishing the diagnosis using the conventional approach resulted in a high rate of inappropriate empirical antibiotic therapy in the non-pneumonia group (46%). Taking into consideration the specificities of patients on VA ECMO is a prerequisite to improve diagnostic performance at the bedside, our team previously showed that the LUS-sCPIS is much more effective than the sCPIS for the early diagnosis of postoperative pneumonia in cardiac patients among whom 22% were under VA ECMO [12]. The presence of a color Doppler intrapulmonary flow within the atelectatic areas (consolidations) is likely characteristic of an inflammatory process, a mechanism that likely explains why the LUS-sCPIS was more discriminating for pneumonia diagnosis. Color Doppler intrapulmonary flow has been reported in community-acquired pneumonia [15, 22], ventilator-associated pneumonia [17], postoperative pneumonia [14], obstructive pneumonia caused by endobronchial tumor [15, 26], and acute respiratory distress syndrome [16, 27].

Few studies have specifically addressed pneumonia in VA ECMO patients [4] and our work is the first to investigate the LUS diagnostic performance. Lung ultrasound is easy to learn [28], provides an immediate and accurate assessment of the patient's respiratory status in acute respiratory failure, and can guide a rapid clinical decision. Incorporating the color Doppler intrapulmonary flow into the LUS-sCPIS yielded an AUC of 0.77, compared with 0.65 for the conventional sCPIS [19]. Confirming a previous statement [11], the dynamic air bronchogram was significantly associated with the diagnosis of pneumonia with a positive predictive value of 95% and a specificity of 96%. The presence of juxta pleural condensation was not discriminating, contrasting with previous studies performed in patients with ventilator-associated pneumonia [11, 29]. In comparison, only the abundant and purulent character of the tracheal secretions was discriminating, against none of the radiological criteria.

The poor performance of the chest radiography is easily explained by the high incidence of bilateral postero-basal consolidations (94% in our study) in mechanically ventilated patients [30]. As previously reported, bilateral radiological infiltrate and coalescent B lines due to hydrostatic pulmonary edema were present in 30% of our patients on ECMO VA [31], complicating the chest radiography interpretation. These results support the concept that combining ultrasound and clinical criteria could be more discriminating than combining radiological and clinical criteria for the diagnosis of pneumonia in patients on VA ECMO.

### Limits

The first limitation of our study is its monocentric nature, with limited included patients albeit the sample size required was achieved. A 26% of VA ECMO patients were heart transplanted. This recruitment bias, linked to the specific characteristics of our center (number one heart transplant center in France), may explain the high rate of pneumonia among included patients (63%) because cardiac transplantation and the postoperative context of cardiac surgery are well-known risk factors for HAP [32]. In addition, the exact prevalence of pneumonia in our cohort of VA ECMO patients is difficult to estimate because not all consecutive patients admitted to our department were included. Only patients with acute respiratory failure have been studied, yet pneumonia is the most frequent cause of acute respiratory failure under VA ECMO. The strategy to prevent the occurrence of HAP in VA ECMO, which involves a bundle of care is described in Supplemental Data (Additional file 4: Table S1). The second limitation is the lack of a gold standard for the diagnosis of HAP, resulting in heterogeneous diagnostic criteria used in most studies that may have led to biases. Despite these elements, the rate of pneumonia found in our study remains comparable to the highest incidences reported in the literature, estimated between 60 and 70% [3, 4, 33]. The sensitivity of color Doppler intrapulmonary flow to detect pneumonia was only 66%, compared with 92% in the study by Dureau et al. [12]. The AUC of LUS-sCPIS was also relatively low (0.77). Physiologically, it is expected that a persisting regional flow within a consolidation will be detected in pneumonia. When lung infection occurs, hypoxic pulmonary vasoconstriction is inhibited by the inflammatory process. When using Doppler during LUS examination, the existence of a color Doppler intrapulmonary flow, defined as the persisting regional flow within the consolidation, is easy to evidence. In VA ECMO patients, the visualization of a color Doppler intrapulmonary flow could be compromised by the modification of transpulmonary blood flow related to the shunting of the cardiac chambers resulting from ECMO. Other parameters may also modify the color Doppler intrapulmonary flow, such as vasoactive support or mechanical ventilation settings. Finally, our study has the biases inherent in its retrospective nature.

### Clinical implication

Any delay in diagnosis and antibiotic treatment of pneumonia in VA ECMO patients, who are already weakened by their critical condition, worsens the prognosis. Conversely, overexposure to unwarranted antibiotic treatment in uninfected patients favors the emergence of bacterial resistance.

Our study shows that in the presence of acute respiratory failure complicating VA ECMO, the detection of a color Doppler intrapulmonary flow and/or a dynamic air bronchogram within a consolidation should alert the clinician to the possible diagnosis of pneumonia. Indeed, posterior consolidations in the critically ill are almost constant and potentially associated with other causes, such as atelectasis. Distinguishing pneumonia from atelectasis in VA ECMO is challenging at the bedside. The only ultrasound sign specific for pneumonia is the dynamic bronchogram [29]. However, it is poorly sensitive and if not visualized, no certain conclusion on the cause of consolidation can be drawn. In particular, if the bronchogram is static or absent, both obstructive atelectasis and pneumonia should be considered. In this situation, the combination of the color Doppler intrapulmonary flow and the dynamic air bronchogram provides additional information. In a previous study about lung ultrasound in cardiac surgery, we have already shown that the combination of air bronchogram and color Doppler intrapulmonary flow for the diagnosis of pneumonia was more accurate than the individual evaluation of these parameters [12]. This result was recently highlighted in a prospective study carried out in ICU [21]. The authors used a decision tree where the LUS-sCPIS was applied after a preselection of patients was made on the presence or not of dynamic air bronchogram and color Doppler intrapulmonary flow. The LUS-sCPIS had a 68% sensitivity and 81% specificity, and its diagnostic accuracy was improved until 86% sensitivity and an 86% specificity when used in a decision tree. This emphasizes the added value of a combination of air bronchogram and color Doppler intrapulmonary flow extended lung ultrasonography assessment to differentiate pneumonia from atelectasis, and help to identify patients who should receive empirical antibiotic therapy after microbiological sampling.

## Conclusion

Our study demonstrates that lung ultrasound is a useful tool as an initial imaging modality for the diagnosis of pneumonia in patients on VA ECMO and is probably more powerful than chest radiography. LUS is rapid and easy to perform at the bedside, in addition to being non-invasive and relatively inexpensive. The presence of a color Doppler intrapulmonary flow visualized using color Doppler is significantly associated with the presence of pneumonia in these patients. LUS-sCPIS is also more accurate than sCPIS in diagnosing pneumonia and may be a useful tool for the appropriate management of cardiac critically ill patients on VA ECMO with a suspected pneumonia.

## Supplementary Information

**Additional file 1.** Movie file of Color Doppler lung ultrasound in the consolidated left lower lobe in a 54 years old patient without pneumonia on veno-arterial extracorporeal membrane oxygenation. Color signals are diffuse and changing resulting from interferences caused by respiratory movements and heart beats. No intrapulmonary shunt is seen.

**Additional file 2.** Movie file of Intrapulmonary shunt detected in the consolidated right lower lobe of a 43 years old patient with pneumonia on veno-arterial extracorporeal membrane oxygenation. The blood flow in a vessel is seen as a color signal persisting in the same location during the respiratory cycle with a tubular, curvilinear, or branching distribution on real-time images. When blood flow signals are detected, pulse-wave Doppler can identify their spectral waveform [19].

**Additional file 3.** Dynamic air bronchogram in a 79 years old patient with pneumonia on veno-arterial extracorporeal membrane oxygenation.

**Additional file 4: Table S1.** Bundle of care to prevent pneumonia in VA ECMO patients. **Table S2.** Lung ultrasound realization: each of the following ultrasound signs was looked for according to this standardized sequence. **Table S3.** sCPIS score as described by Luna et al [17]. **Table S4.** LUS-sCPIS score as described by Dureau et al [10]. The criterion based on the interpretation of the chest radiograph is the original sCPIS score is replaced by the presence or absence of a shunt on color Doppler. **Table S5.** comparison of the two groups, with and without pneumonia, according to the individualized criteria of the sCPIS score. Categorical variables are expressed as headcount (%).

## Data Availability

The datasets used and/or analyzed during the current study are available from the corresponding author on reasonable request.

## Abbreviations

AUC: Area under the curve
BAL: Bronchoalveolar lavage
BMI: Body mass index
CPIS: Clinical Pulmonary Infection Score
FiO_2_: Fraction of inspired oxygen
FmO_2_: Fraction of delivered oxygen in the sweep gas on VA ECMO
HAP: Hospital-acquired pneumonia
LUS: Lung ultrasound
LVAD: Left ventricular assist device
PCT: Procalcitonin
PTP: Plugged telescopic catheter
Se: Sensitivity
sCPIS: Simplified Clinical Pulmonary Infection Score
SIRS: Systemic inflammatory response syndrome
SOFA: Sepsis-related Organ Failure Assessment
Sp: Specificity
LUS-sCPIS: Lung ultrasound-simplified Clinical Pulmonary Infection Score, where the "chest radiography" criterion is replaced by the presence or not of a color Doppler intrapulmonary flow within a consolidation, visualized using color Doppler
NPV: Negative predictive value
PPV: Positive predictive value
VA ECMO: Venoarterial extracorporeal membrane oxygenation
