# Supplementary material for: Usefulness of lung ultrasound for early detection of hospital-acquired pneumonia in cardiac critically ill patients on venoarterial extracorporeal membrane oxygenation
Source: Ann Intensive Care. 2022 May 21;12:43. doi: 10.1186/s13613-022-01013-9 (PMC9124275; doi:10.1186/s13613-022-01013-9)
Supplement: Supplementary file 4 — Additional file 4: Table S1. Bundle of care to prevent pneumonia in VA ECMO patients. Table S2. Lung ultrasound realization: each of the following ultrasound signs was looked for according to this standardized sequence. Table S3. sCPIS score as described by Luna et al [17]. Table S4. LUS-sCPIS score as described by Dureau et al [10]. The criterion based on the interpretation of the chest radiograph is the original sCPIS score is replaced by the presence or absence of a shunt on color Doppler. Table S5. comparison of the two groups, with and without pneumonia, according to the individualized criteria of the sCPIS score. Categorical variables are expressed as headcount (%). [file 13613_2022_1013_MOESM4_ESM.docx]

**Diagnostic performance of Color Doppler Lung Ultrasound in the Diagnosis of Hospital-Acquired Pneumonia in Patients Assisted with Veno-Arterial Extra Corporeal Membrane Oxygenation.**

***Supplementary Files***

**Table S1:** Bundle of care to prevent pneumonia in VA ECMO patients

| **For all patients:**   - Elevation of head of bed (30°–45°) within the limit authorized by the positioning of the ECMO cannulas - Provide excellent oral hygiene care. - Early exercise and mobility, particularly pulmonary rehabilitation - Apply conservative fluid management, and conservative blood transfusion thresholds |
| --- |
| **In case of mechanical ventilation:**   - Use of subglottic secretion drainage in case of mechanical ventilation - Use protective ventilation strategy in case of mechanical ventilation - Daily sedation interruption and assessment of readiness to extubate - Early liberation from mechanical ventilation |
| **Specific for ECMO patients:**   - Daily cardiac evaluation and assessment of readiness to wean VA ECMO - Weaning from ECMO as soon as possible |

**Table S2:** Lung ultrasound realization: each of the following ultrasound signs was looked for according to this standardized sequence.

| **Lung sliding**   - Visualization of the pleural line: horizontal hyperechoic line, mobile, visible about 0.5 cm below the ribs. |
| --- |
| **Juxta pleural consolidation**   - Presence of hypoechoic tissue, adjacent to the pleura, with a diameter greater than 5mm, from which B lines are derived. |
| **Consolidation**   - **Static or dynamic air bronchogram:** punctiform or linear hyperechoic images within a consolidation. The bronchogram is dynamic if these same images are characterized by an inspiratory centrifugal movement. - **Presence of a shunt:** color Doppler visualization of persistent regional pulmonary blood flow |
| **Lung ultrasound aeration score**  *Sum of the score in each of the 12 quadrants, total /36.*   - 0 = normal aeration (lung sliding with horizontal A lines, fewer than 2 isolated vertical B lines) - 1 = moderate loss of lung aeration: presence of multiple well-defined and spaced B1 lines issued from the pleural line or from small juxtapleural consolidations, in a limited portion of an intercostal space. - 2 = severe loss of lung aeration: multiple coalescent vertical B2 lines issued either from the pleural line or from juxtapleural consolidations, detected in the whole quadrant or several intercostal spaces. - 3 = consolidation |
| **Presence of a pleural effusion** |

**Table S3:** sCPIS score as described by Luna et al ^17^*.*

|  | Variables | Points |
| --- | --- | --- |
| Temperature | ≥ 36.5 and ≤ 38.4  ≥ 38.5 and ≤ 38.9  ≥ 39.0 and ≤ 36.0 | 0  1  2 |
| Leucocytes /mm^3^ | ≥ 4000 and ≤ 11 000  < 4000 or > 11 000 | 0  1 |
| Tracheal secretions | Few  Moderate  Abundant  Purulent | 0  1  2  +1 |
| Oxygenation | PaO2/FiO2 > 240 or presence of ARDS  PaO2/FiO2 ≤ 240 and absence of ARDS | 0  2 |
| Chest X-rays | No infiltrates  Patchy or diffuse infiltrate  Localized infiltrate | 0  1  2 |

**Table S4:** LUS-sCPIS score as described by Dureau et al ^10^. The criterion based on the interpretation of the chest radiograph is the original sCPIS score is replaced by the presence or absence of a shunt on color Doppler.

|  | Variables | Points |
| --- | --- | --- |
| Temperature | ≥ 36.5 et ≤ 38.4  ≥ 38.5 et ≤ 38.9  ≥ 39.0 et ≤ 36.0 | 0  1  2 |
| Leucocytes /mm^3^ | ≥ 4000 et ≤ 11 000  < 4000 ou > 11 000 | 0  1 |
| Tracheal secretions | Rare  Moderate  Abundant  Purulent | 0  1  2  +1 |
| Oxygenation | PaO2/FiO2> 240 or presence of d'un ARDS  PaO2/FiO2≤ 240 and absence of ARDS | 0  2 |
| Color Doppler LUS | No shunt  Shunt | 0  2 |

**Table S5**: comparison of the two groups, with and without pneumonia, according to the individualized criteria of the sCPIS score. Categorical variables are expressed as headcount (%).

|  | **Total (n=70)** | **Pneumonia** | | **P** |
| --- | --- | --- | --- | --- |
|  |  | **No (n=26)** | **Yes (n=44)** |  |
| **Leucocyte count** |  |  |  | 0.51 |
| ≥ 4000 and ≤ 11 000 | 25 (36) | 8 (31) | 17 (39) |  |
| < 4000 or > 11 000 | 45 (64) | 18 (69) | 27 (61) |  |
| **Temperature** |  |  |  | 1 |
| ≥ 36.5 and ≤ 38.4 | 52 (74) | 19 (73) | 33 (75) |  |
| ≥ 38.5 and ≤ 38.9 | 3 (4.3) | 1 (3.8) | 2 (4.5) |  |
| ≥ 39.0 and ≤ 36.0 | 15 (21) | 6 (23) | 9 (20) |  |
| **Tracheal secretions** |  |  |  | **< 0.01** |
| Rare | 25 (36) | 15 (58) | 10 (23) |  |
| Moderate | 17 (24) | 6 (23) | 11 (25) |  |
| Abundant | 28 (40) | 5 (19) | 23 (52) |  |
| **Purulent secretion** | 24 (34) | 5 (19) | 19 (43) | **0.041** |
| **Chest X ray** |  |  |  | 0.32 |
| No infiltrate | 13 (19) | 3 (12) | 10 (23) |  |
| Diffuse infiltrate | 43 (61) | 19 (73) | 24 (55) |  |
| Focal infiltrate | 14 (20) | 4 (15) | 10 (23) |  |
